# Develop a circular RNA–related regulatory network associated with prognosis of gastric cancer

**DOI:** 10.1002/cam4.3035

**Published:** 2020-09-09

**Authors:** Shuxun Wei, Shifeng Teng, Jun Yao, Wenchao Gao, Jia Zang, Guangyong Wang, Zhiqian Hu

**Affiliations:** ^1^ Department of General Surgery The Second Military Medical University/Changzheng Hospital Shanghai China; ^2^ Department of Gastroenterology The Second Military Medical University/Changhai Hospital Shanghai China

**Keywords:** ceRNA, circRNA, gastric cancer, prognosis, regulatory network

## Abstract

**Background:**

In gastric cancer (GC), circular RNAs (circRNAs) mainly play an important role in miRNA sponge, which not only indicate long‐term survival and prognosis but also increase resistance to the apoptosis. The purpose of the study is to explore new circRNAs and their underlying mechanisms in GC.

**Method:**

Through rigorous retrieval strategies, we used the sva package to analyze and identify differentially expressed circRNAs (DECs) from three Gene Expression Omnibus microarray datasets (GSE83521, GSE89143, and GSE78092). Online website CSCD and CircInteractome were used to reveal the binding sites between miRNAs and DECs. The possible target miRNAs of the DECs identified based on miRNAs, and Cytoscape was used to create a regulatory network of circRNA‐miRNA‐mRNA and identified the hub genes which were further validated using The Cancer Genome Atlas database and Human Protein Atlas.

**Results:**

Twenty‐eight DECs were obtained using the sva package. A regulatory network of circRNA‐miRNA‐mRNA (competing endogenous RNA) containing 15 circRNAs, 24 miRNAs, and 158 genes was identified. A protein‐protein interaction network based on the 158 genes was established, and further determined that 10 hub genes (SKA1, ANLN, CHEK1, SKA3, TOP2A, BIRC5, RRM2, NCAPG2, FANCI, and RAD51) were associated with some cancer‐related pathways based on the functional enrichment analysis. Finally, six hub genes (BIRC5, TOP2A, FANCI, NCAPG2, RAD51, and RRM2) were proven to influence the overall survival of GC.

**Conclusion:**

Our study established a circRNA‐miRNA‐mRNA regulatory network and defined six circRNA‐related hub genes in GC, which could serve as potential therapeutic targets or prognostic biomarker for GC treatment.

## Graphical abstract

Gene Ontology and Kyoto Encyclopedia of Genes and Genomes enrichment annotations of differentially expressed genes (DEGs) (up‐(A) and down (B)‐regulated DEGs).

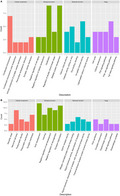

## 1 INTRODUCTION

As one of the most common human aggressive malignancies, gastric cancer (GC) is the second cause of cancer‐related mortality across the world.^1^, ^2^ Despite great development in the diagnosis and treatment of the disease, the prognosis in patients with GC remains poor,^3^, ^4^ due to the limited treatment options in both original advanced and metastatic stage.^5^ Therefore, it is urgent to explore the potential mechanisms and therapeutic targets for GC, which could reduce the severity of GC. Increasing studies have revealed that patients’ outcomes do not only depend on staging but also on specific molecules, and recently, the critical role of circular RNA (circRNA) in the underlying mechanisms of the occurrence and progression of GC have attracted much more attention.^6^, ^7^

With high stability, biological evolutionary conservatism, and tissue expression specificity, circRNA is a widely present in endogenous non‐coding RNA and has important biological functions such as playing the role of miRNA sponge, regulating the expression of a gene in patient, and improving transcription and translation levels that are related to transcriptional and posttranscriptional regulation of gene expression.^8^, ^9^, ^10^ CircRNA_001569 promoted the proliferation of GC cells through absorbing miR‐145,^11^ and CircPSMC3, as a competitive endogenous RNA, inhibited the development and metastasis of GC cells by sponging miR‐295‐5p.^12^ Also, circRNA_104916 regulated apoptosis, metastasis, and epithelial‐mesenchymal transition^13^ in colon cancer cells. In gastrointestinal tumor, circRNA mainly plays the role of miRNA sponge, which affects the proliferation, differentiation, apoptosis, and invasion of cancer cells.^6^, ^7^

The newly advanced high‐throughput RNA sequencing technologies turned circRNA back to the field of biological research with their precise detection and investigation.^14^ Therefore, in order to further seek the underlying effect of circRNAs in GC, differentially expressed circRNAs (DECs) were identified by a microarray. Then, we developed a regulatory circRNA‐miRNA‐mRNA network based on our datasets. To find the downstream targets of the regulatory network, we used Kaplan‐Meier analysis to identify six hub genes which were closely associated to the overall survival (OS) of patients with GC. The six hub genes (BIRC5, TOP2A, FANCI, NCAPG2, RAD51, and RRM2) were reported to be critical to regulate cell cycle, and their abnormal expression could lead to development and progression of cancers.^15^, ^16^, ^17^, ^18^, ^19^ This study would present a new regulatory network underlying GC pathogenesis and provide novel biomarkers and targets for GC diagnosis and treatment.

## 2 MATERIALS AND METHODS

### 2.1 Selection of patients with GC

According to the detailed retrieval strategy (Supporting Information 1), a total of eight microarray datasets for circRNA expression profiles related to GC were downloaded from Gene Expression Omnibus (GEO, https://www.ncbi.nlm.nih.gov/geo/). After filtering based on the inclusion and exclusion criteria (Supporting Information 1), a total of three datasets (GSE83521, GSE89143, and GSE78092) were acquired from the above eight datasets. In addition, the level 3 RNA‐sequencing data and corresponding clinical information for patients with gastric adenocarcinoma (STAD) were downloaded from TCGA (The Cancer Genome Atlas) by the TCGAbiolinks package of R software (Version 2.8.4).

### 2.2 Identification of DECs and genes in GC

The above three datasets screened for circRNA profiles were downloaded through *R* package *GEOquery* and converted into log_2_ form. The *sva* package was used to decrease the batch effect and merge the three datasets. Based on the screened thresholds of |log_2_(fold change)| > 1 and *P* < 0.05, DECs in each dataset were further defined by using *limma* package. Similarly, to obtain the differentially expressed genes (DEGs), the level 3 RNA‐sequencing data for mRNA profile of STAD were calculated by *DESeq2* package based on the same screened criteria.

### 2.3 Prediction of MREs

The prediction of binding sites between miRNAs and those selected DECs, were performed by two web tools, cancer‐specific circular RNAs (CSCD, http://gb.whu.edu.cn/CSCD/) and Circular RNA Interactome (CircInteractome, https://circinteractome.nia.nih.gov/). The underlying binding miRNAs of the DECs were identified by the overlapped miRNAs to develop circRNA‐miRNA interactions.

### 2.4 Target genes of miRNA

The miRNA‐mRNA interactions were constructed by *miRWalk 3.0* (http://mirwalk.umm.uni‐heidelberg.de/), which involves three predicted algorithms (Targetscan, miRDB, and miRTarBase)., Target genes retrieved from the three databases should meet the following two criteria: (a) defined target genes were predicted by at least one algorithm; and (b) the predicted score of target genes must be >0.95. The score derived from TarPmiR algorithm can be used to predict miRNA target site. Genes shared by the predicted genes and DEGs were selected as candidate genes to construct network related to competing endogenous RNA (ceRNA) network.

### 2.5 Construction of ceRNA network

According to the predicted target interactions between circRNA, miRNA, and mRNA, the ceRNA network was finally determined based on the same expression trend between circRNA and its corresponding mRNA. The Cytoscape software was used to visualize the regulatory network of ceRNA.

### 2.6 Functional enrichment analysis

Based on the DEGs in the preliminary ceRNA regulatory network, the further analysis of Gene Ontology (GO) and Kyoto Encyclopedia of Genes and Genomes (KEGG) was performed by the *cluster Profiler* package. The top five GO and KEGG items with the most meaningful are also displayed by this package, respectively.

### 2.7 Protein‐protein interaction Network analysis and hub genes

The online website *STRING* (https://string‐db.org/) was used to develop a protein‐protein interaction (PPI) network based on the target genes in ceRNA regulatory network. We generated the PPI network based on seven interaction sources using a medium confidence (0.400) interaction score. Then, with the Molecular Complex Detection plug‐in, Cytoscape was employed to analyze PPI network and further search for the hub genes. In addition, centiscape (version 2.2) plug‐in was used to calculate the degree of every target genes in the PPI network.

### 2.8 Examination and survival analysis of hub genes in GC

The verification of hub genes defined on the protein level was performed by the website of *Human Protein Atlas* (https://www.proteinatlas.org/). In addition, to assess the prognostic value of the hub genes, the influence of hub genes on survival of GC patients were also analyzed based on the level 3 RNA‐sequencing data of STAD. To make the results more reliable, the data with survival time <30 days were excluded^20^, ^21^ and the mean values were used to divide gene expression into high and low levels.

## 3 RESULTS

### 3.1 Identification of DECs in GC based on sva package

**FIGURE 1:**
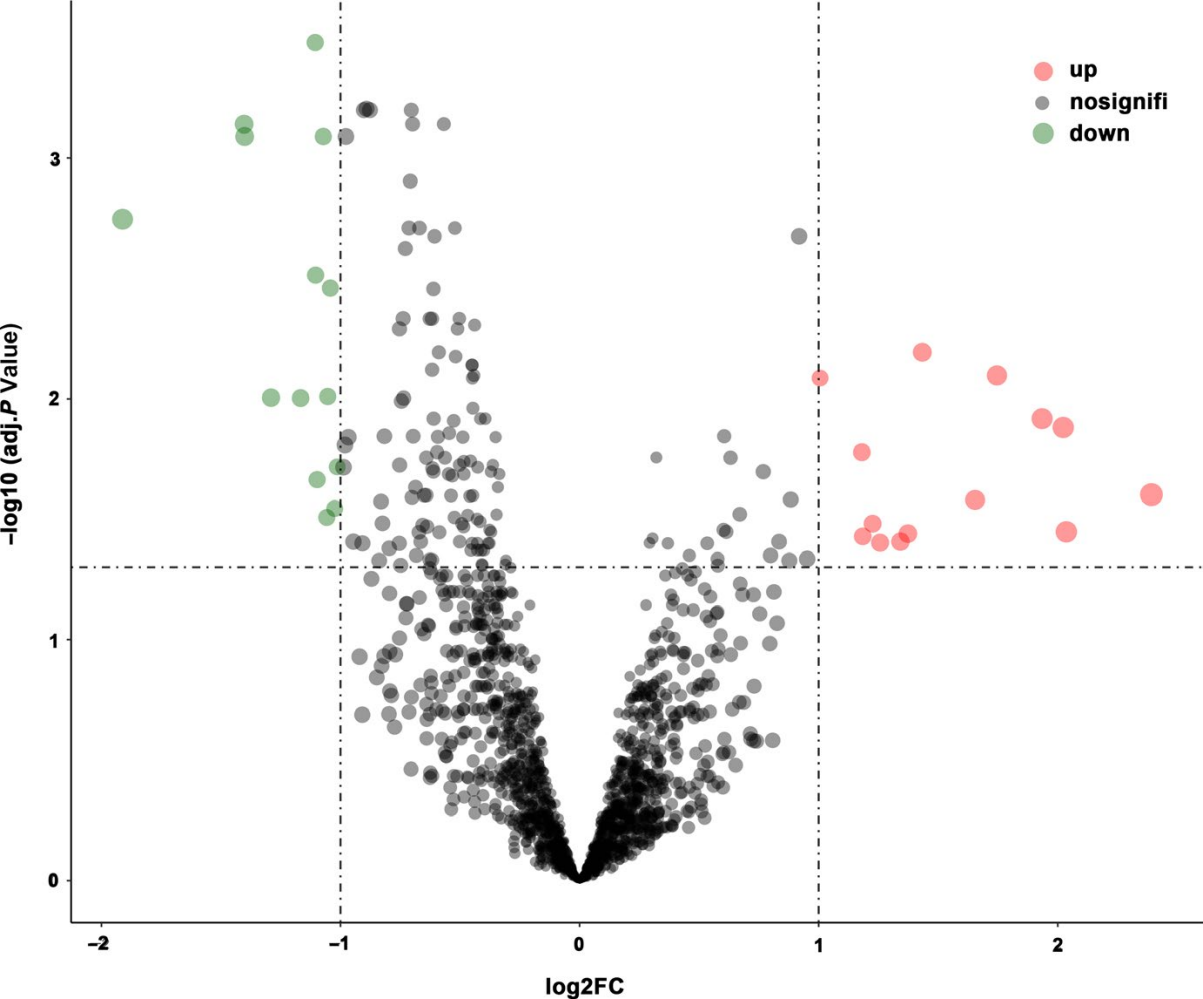
Volcano plots for differentially expressed circRNAs identified from Gene Expression Omnibus

**FIGURE 2:**
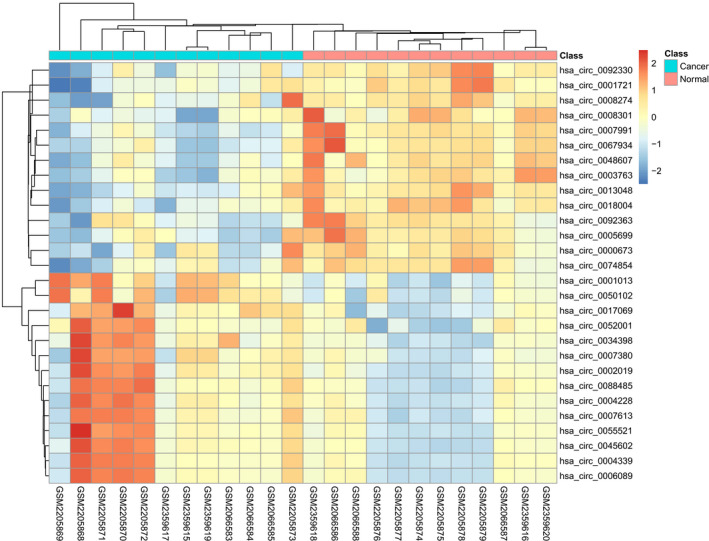
Heatmap for the 28 differentially expressed circRNAs in three microarray datasets (GSE83521, GSE89143, and GSE78092)

According to the inclusion and exclusion criteria, three high‐quality microarray datasets (GSE83521, GSE89143, and GSE78092) were included in our study (Figure S1; Supporting Information 2). Gene chips of GSE83521 (six cancer tissues and six paracancer tissues) and GSE89143 (three cancer tissues and three paracancer tissues) were obtained from the platform of Agilent‐069978 Arraystar Human CircRNA microarray V1.0, and the chip of GSE78092 (three cancer tissues and three paracancer tissues) was based on ArrayStar Human Circular RNA microarray V2.0. The information of included datasets is shown in Table S1. This research explored the circRNA expression levels in a total of 12 cancer tissues and 12 normal tissues derived from GC patients. The experimental design is shown in Figure S2. A total of 28 DECs were identified in the integrated dataset, including 14 upregulated circRNAs and 14 downregulated circRNAs. Based on the two dimensions of −log_10_ (FDR) and log_2_(FC), the distribution of all DECs are displayed by a volcano map (Figure 1). The specific expression of the screened DECs was demonstrated by the heatmap as shown in Figure 2.

### 3.2 Prediction of circRNA‐miRNA pairs

With the miRNA‐sponge function, circRNAs can bind and suppress its corresponding miRNAs, thus affecting the expression levels of its downstream mRNAs.^8^ Therefore, in order to predict the potential target miRNAs of all DECs, two online databases (CSCD and CircInteractome) were used to analyze the data based on the above select condition, and a total of 37 pairs including 19 circRNAs and 31 miRNAs were determined from the two databases.

### 3.3 Prediction of miRNA‐mRNA pairs

**FIGURE 3:**
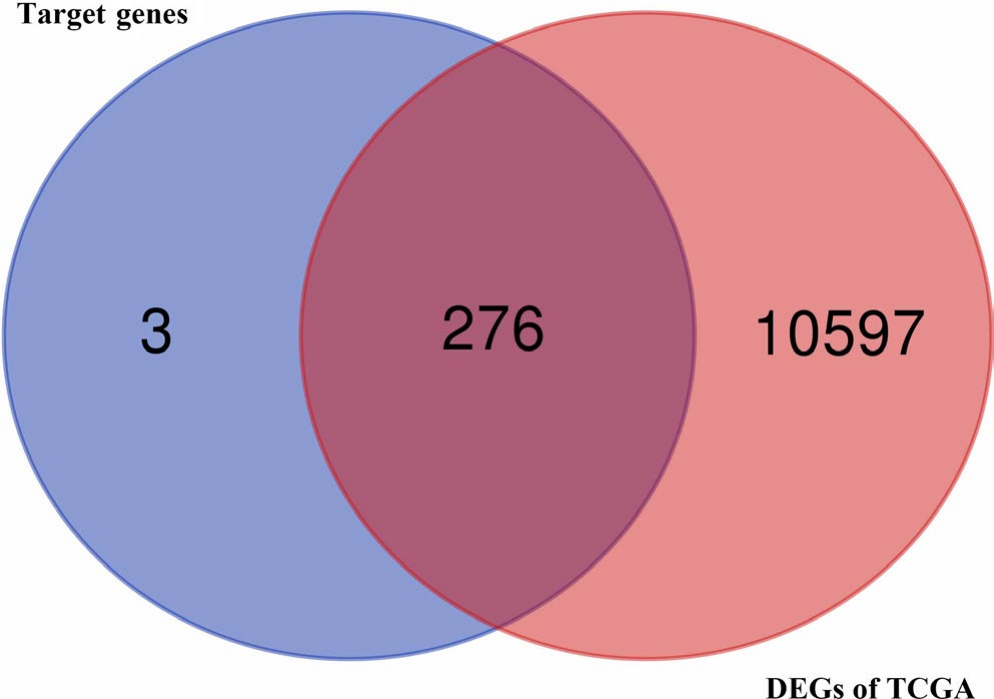
Venn diagram for the intersections between DEGs of TCGA and miRNA target genes. DEG, differentially expressed gene; TCGA, The Cancer Genome Atlas

It is that miRNAs could inhibit the transcription or promote the degradation of mRNAs via interacting with the target mRNAs directly.^22^, ^23^ For the purpose to forecast the target genes of the above 31 miRNAs, 1647 target genes were obtained by using the tool of miRWalk. Besides, 10 873 DEGs in GC were obtained from the TCGA dataset of STAD. Finally, a total of 279 duplicated genes between the target genes and DEGs were included in the final miRNA‐mRNA pairs (Figure 3, Supporting Information 3).

### 3.4 Construction of the ceRNA network

**FIGURE 4:**
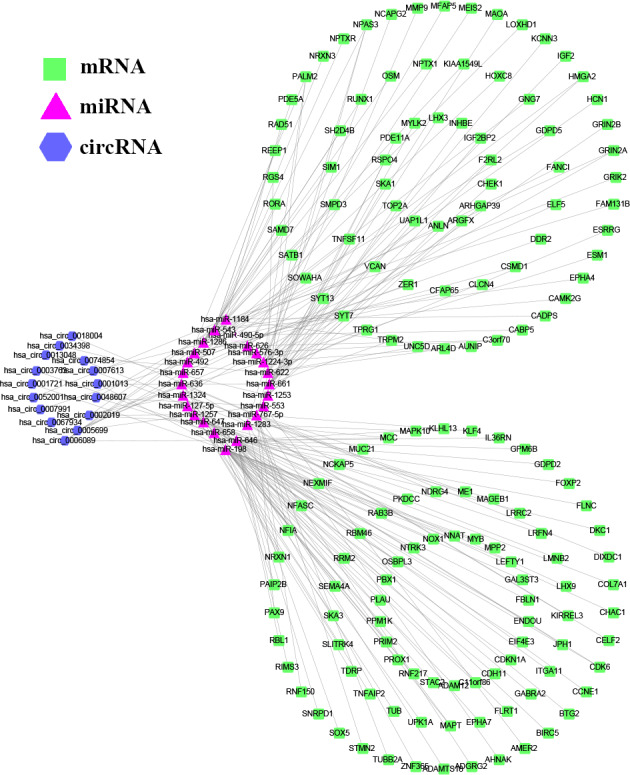
circRNA‐miRNA‐mRNA regulatory network. The network consisting of 15 circRNAs, 24 miRNAs, and 158 genes was generated by Cytoscape 3.6.1

In order to further explore the effect of circRNA on mRNA via binding miRNA in GC, an integrated ceRNA network was developed by combining the pairs of circRNA‐miRNA and miRNA‐mRNA, according to the similar expression trend of circRNA and its corresponding mRNA (Figure 4). Finally, there are 15 circRNAs, 24 miRNAs, and 158 genes that were identified as differentially expressed profiles in the regulatory ceRNA network, which were visualized by using Cytoscape.

### 3.5 Functional analysis of DEGs in ceRNA regulatory network

**FIGURE 5:**
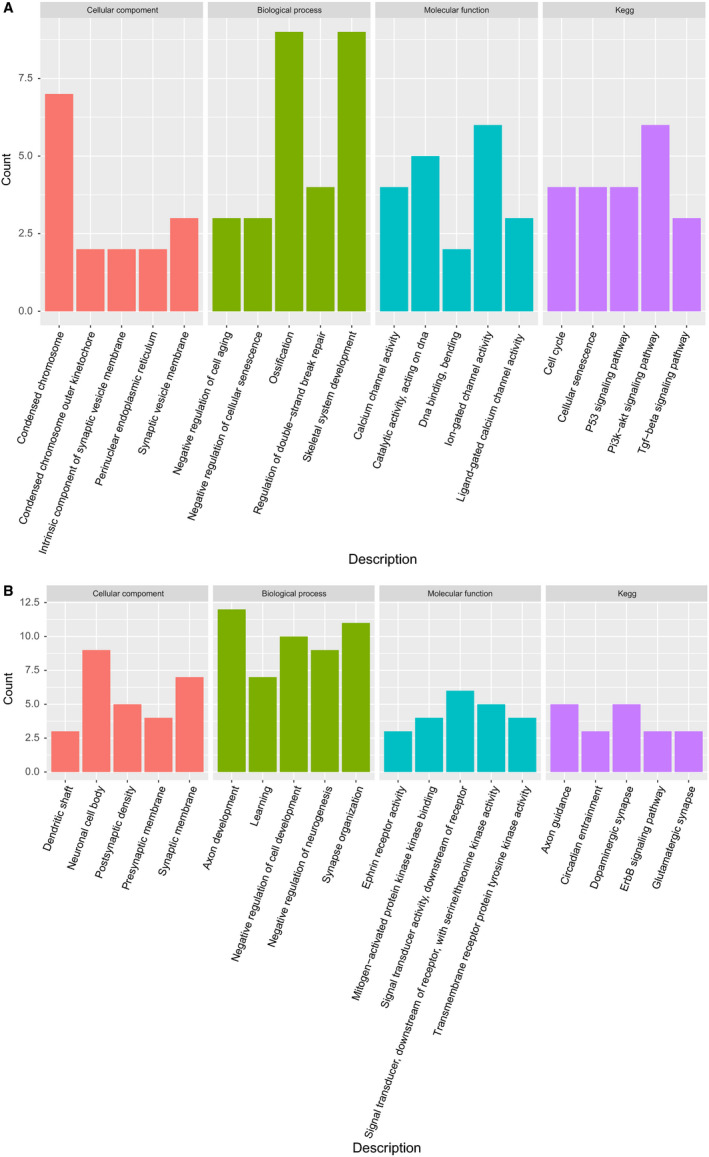
GO and KEGG enrichment annotations of DEGs (up‐(A) and down (B)‐regulated DEGs). DEG, differentially expressed gene; GO, Gene Ontology; KEGG, Kyoto Encyclopedia of Genes and Genomes

In order to further explore the underlying mechanisms involved in GC development, the cluster Profiler package was used to analyze the biological function of the 158 genes in regulatory network via the enrichment analysis of GO and KEGG; enrichment results of GO and KEGG of the up‐ and downregulated genes are shown in Figure 5A,B, respectively. The most enriched GO terms related to upregulated genes in cellular component (CC) were “condensed chromosome, perinuclear endoplasmic reticulum, and condensed chromosome outer kinetochore,” in biological process (BP) were “ossification, negative regulation of cellular senescence, and regulation of double‐strand break repair,” and that in molecular function (MF) were “ligand‐gated calcium channel activity, catalytic activity, and acting on DNA.” The KEGG pathways related to upregulated genes were mainly concentrated in the pathways of p53 signaling pathway, cell cycle, and PI3K‐Akt signaling pathway (Supporting Information 4).

Considering the downregulated genes, the most enriched GO terms related to upregulated genes in CC were “neuronal cell body, presynaptic membrane, and synaptic membrane,” that in BP were “synapse organization, negative regulation of cell development, and axon development,” and that in MF were “ephrin receptor activity, signal transducer activity‐downstream of receptor, signal transducer, and downstream of receptor‐with serine/threonine kinase activity.” The KEGG pathways related to upregulated genes were mainly concentrated in the pathways of dopaminergic synapse, axon guidance, and amphetamine addiction (Supporting Information 5).

The above GO terms and KEGG pathways were involved in cancer occurrence and development to some extent, especially the upregulated DEGs which were majorly associated with cell cycle and DNA damage repair.

### 3.6 Construction of PPI network

**FIGURE 6:**
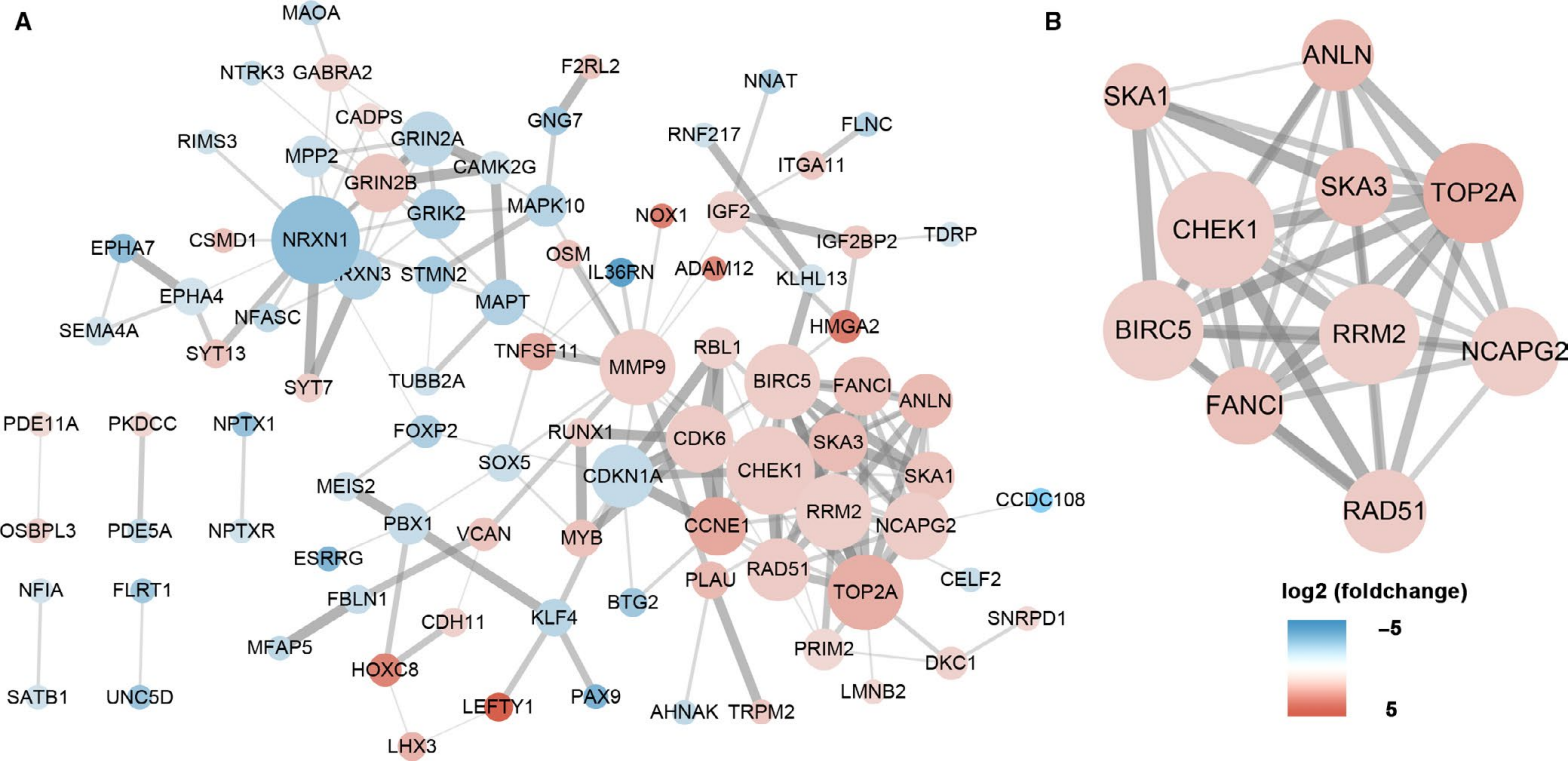
Identification of hub genes from the PPI network with the MCODE algorithm. The node color changes gradually from blue to red according to the log2(foldchange) of genes. The node size changes gradually from small to big according to the degree calculated by the Centiscape plug‐in. The edge size represents the strength between two node which calculated by online website STRING. A, A PPI network of the 158 target genes. B, A PPI network of the 10 hub genes. PPI, protein‐protein interaction

To further better understand the 158 genes predicted in GC. The PPI network of the 158 overlapped genes was established based on the STRING database (Figure 6A). The original network contained 90 nodes and 178 edges. By utilizing the algorithm of MCODE, the highest score the subnetwork was determined (Figure 6B). A total of 10 genes (SKA1, ANLN, CHEK1, SKA3, TOP2A, BIRC5, RRM2, NCAPG2, FANCI, and RAD51) in this subnetwork are defined as hub genes. These hub genes are all included in the upregulated DEGs in ceRNA regulatory network.

### 3.7 Survival analysis of hub genes in GC patients

**FIGURE 7:**
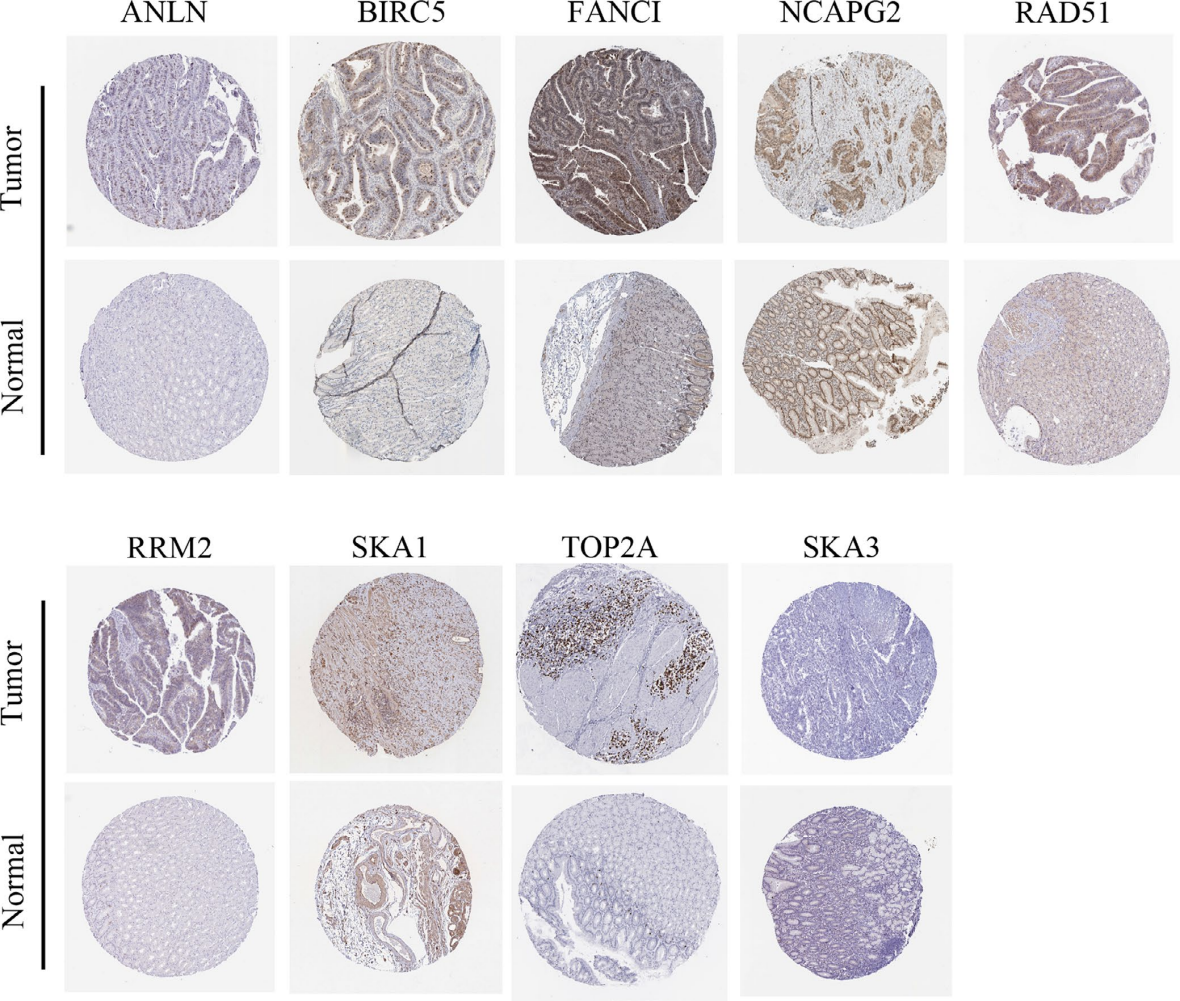
Images of immunohistochemical staining with the 10 hub genes

**FIGURE 8:**
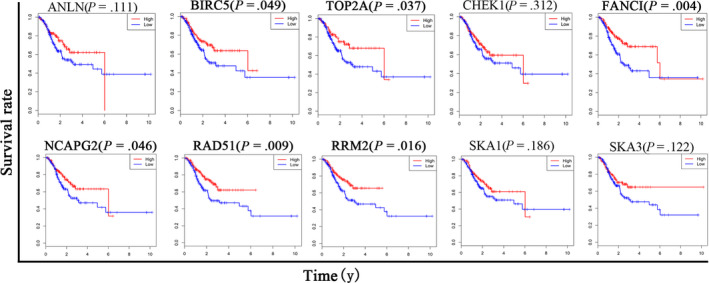
Survival curves of the 10 hub genes

To further explore the expression status of the hub genes in GC, the protein levels of the hub genes were displayed in tumor tissues and normal tissues, respectively, according to the analysis based on Human Protein Atlas database (Figure 7), which revealed that most of the hub genes tended to express higher in tumor than the normal tissues in most of the patients with GC. Moreover, to validate the influence of hub genes on GC prognosis, Kaplan‐Meier curve analysis was performed to analyze the correlation between the expression of the 10 hub genes (SKA1, ANLN, CHEK1, SKA3, TOP2A, BIRC5, RRM2, NCAPG2, FANCI, and RAD51) and the OS of patients with GC. According to the analysis of TCGA data of STAD, the result showed that six hub genes (BIRC5, TOP2A, FANCI, NCAPG2, RAD51, and RRM2) among the above hub genes influenced the OS of GC significantly (Figure 8).

## 4 DISCUSSION

As the fourth most incident and second leading cause of cancer‐related death worldwide, GC was developed due to the accumulation of multiple genetic alterations in gastric carcinogenesis.^4^, ^5^ However, the underlying mechanisms that affect its tumorigenesis and progression remain unclear, especially in the field of circRNAs.

With the wide application of high‐throughput RNA sequencing technologies in cancer‐related basic biomedical research, circRNAs, a class of non‐coding RNAs, attracted amount of interest and attention gradually, which play critical role in miRNA sponges, gene transcription regulation, RNA‐binding proteins, and their translation thus to affect the expression levels of many oncogenes.^8^, ^24^ Since circRNAs play critical role in miRNA sponges, gene transcription regulation, RNA‐binding proteins, and their translation of many oncogenes, they attracted amount of interest and attention gradually.^8^, ^24^, ^25^ In the present study, we analyzed all the circRNA datasets of GC in the GEO database to explore the potential underlying mechanism of circRNA‐associated miRNA sponge in GC development. Also. increasing studies have revealed that circRNA mainly functions as a miRNA sponge, thus to affect the biological functions of GC cells.^10^, ^25^, ^26^

Especially in recent years, increasing studies have revealed that circRNA mainly functions as a miRNA sponge, which affects the proliferation, differentiation, apoptosis, and invasion of GC cells.^10^, ^26^ In our study, first, 28 DECs (14 upregulated and 14 downregulated circRNAs) were identified. A quite vital and functional aspect of circRNA was its sponge activity with miRNA when localized in the cytoplasm.^8^ Based on the online tool miRWalk (version 3.0), 279 repeated genes between the predicted genes and DEGs of STAD were collected, and a ceRNA regulatory network containing 15 circRNAs, 24 miRNAs, and 158 genes was constructed.

In addition, the GO and KEGG analysis indicated that those genes were related to various important BP associated with tumors, which play a critical part in the tumorigenesis and development of various tumors. Therefore, these molecules included circRNAs, miRNAs, and genes in the ceRNA network could be involved in the prognosis and development of GC.

Moreover, 10 hub genes were defined based on the PPI network, and further analyzed to evaluate their effect on GC prognosis in TCGA database. Previous studies have reported the potential mechanisms of SKA1, ANLN, RRM2, and RAD51 in the carcinogenesis of GC. Sun et al found that SKA1 could be used as a biomarker for GC early diagnosis,^27^ and our study also confirmed SKA1 may be identified as a prognostic biomarker for GC. ANLN is a critical predictor for GC by regulating Wnt/β‐catenin signaling pathway.^28^ Zhong et al found that the RRM2 can influence the invasiveness of GC cells by RM2/AKT/NF‐κB pathway.^29^ Another study showed RAD51 potentiated the synergistic effects of chemotherapy on GC.^30^

It should be noted that the expression of hub genes was validated on the protein level. The protein expression trend was the same as the mRNA level trend of most of the hub genes. However, the immumohistochemical staining of CHEK1 was not found, and the immumohistochemical staining of SKA3 showed low expression in cancer tissues of patients with GC, which was different from the trend of its mRNA expression.

Furthermore, the result demonstrated that only six hub genes (BIRC5, TOP2A, FANCI, NCAPG2, RAD51, and RRM2) influenced the OS of GC significantly, which majorly participated in the regulation of cell cycle. BIRC5 is an evolutionarily conserved eukaryotic protein that is essential for cell mitosis and can inhibit cell death.^31^, ^32^ Catenations of DNA formed during replication are decatenated by TOP2A in G2 phase of cell cycle.^33^ As a rate‐limiting enzyme for DNA synthesis and repair, downregulation of RRM2 caused cell arrest in the G0/G1 phase and promoted cell apoptosis,^34^ whose overexpression could promote the invasiveness of GC cells via AKT/NF‐κB signaling pathway.^29^ NCAPG2 plays a critical role in cell mitosis,^17^ and the inhibition of RAD51, participating in DNA damage repair, induced the G2/M phase arrest.^35^ Moreover, RAD51 could potentiate synergistic effects of chemotherapy with PCI‐24781 and *cis*‐diamminedichloroplatinum on GC cells.^30^ In addition, FANCI and NCAPG2 have not yet been studied in GC. Therefore, the hub genes may have critical role in GC progression and should be further studied in GC in the future, especially the two hub genes FANCI and NCAPG2. Our paper uses a different method to obtain DECs, which further confirmed the vital role of cell cycle‐related hub genes in development and progression of GC.

This study has its own limitations. (a) The number of patients enrolled in this study is still not enough, and more reliable results would be obtained from a larger sample size. (b) Our study was mainly based on the bioinformatics calculation method, which was not verified by experiments. Thus, the abnormal expression and function of the hub genes should be confirmed in the future study.

In summary, our study developed a ceRNA regulatory network to explore the potential circRNAs‐related mechanisms in GC, and defined the circRNAs‐related hub genes, which may serve as potential novel therapeutic targets.

## CONFLICT OF INTEREST

The authors declare no conflict of interest.

## AUTHOR CONTRIBUTIONS

ZH and GW are responsible for the study design and interpretation. SW, ST, and JY collected and analyzed the data. SW, ST, WG, and JZ assisted in interpretation and preparation of the manuscript. All authors have read and approved the manuscript.

## ETHICS APPROVAL AND CONSENT TO PARTICIPATE

None.

## CONSENT FOR PUBLICATION

Not applicable.

## Supporting information

Figure S1

Figure S2

Table S1

Supplementary Material

Supplementary Material

Supplementary Material

Supplementary Material

Supplementary Material

Supplementary Material

## Data Availability

The GEO database (https://www.ncbi.nlm.nih.gov/geo/) are public available.
