# Supplementary figures and images for: Develop a circular RNA–related regulatory network associated with prognosis of gastric cancer
Source: Cancer Med. 2020 Sep 9;9(22):8589–99. doi: 10.1002/cam4.3035 (PMC7666747; doi:10.1002/cam4.3035)

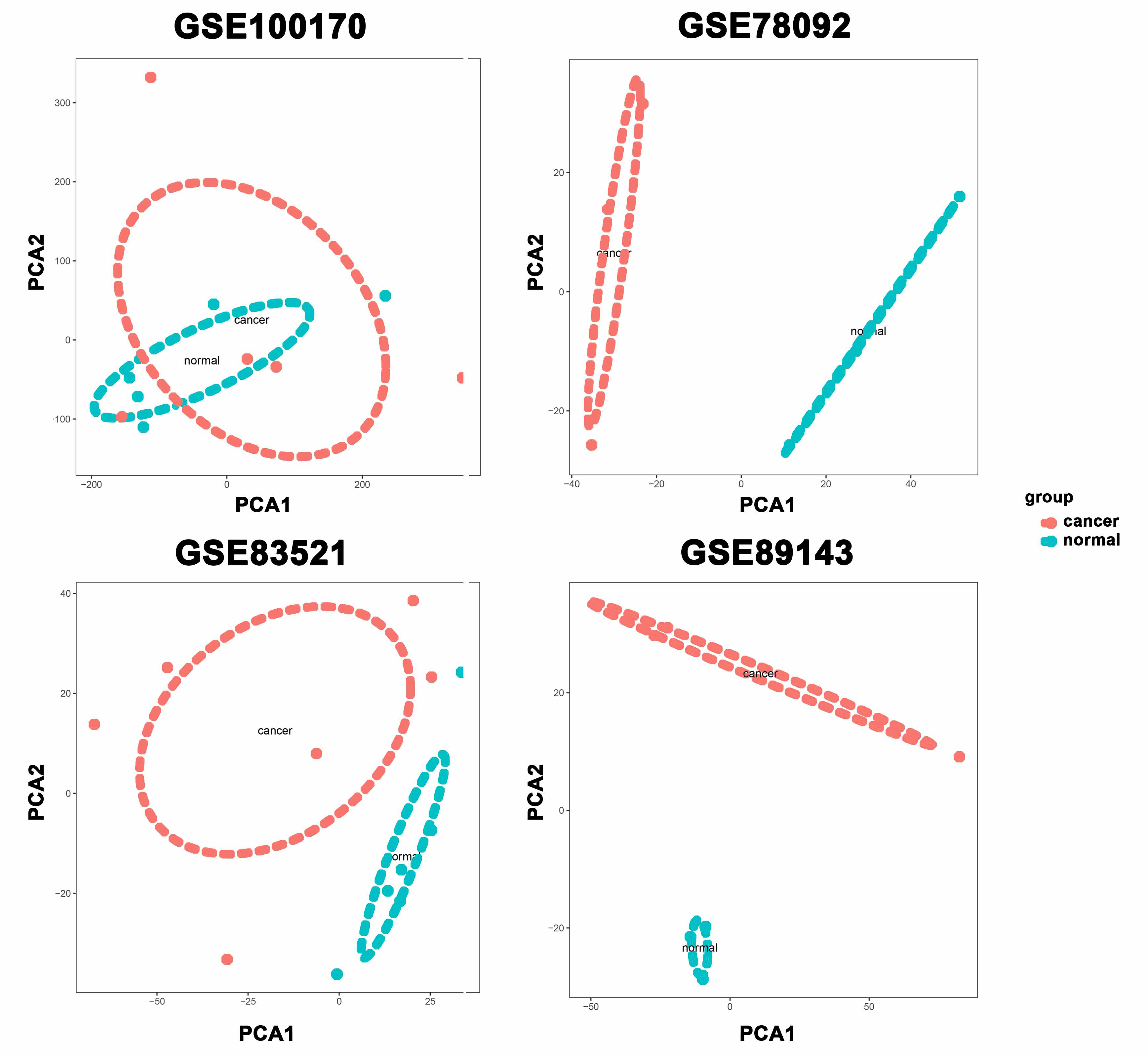

Supplement: Supplementary file 1 — Figure S1 [file CAM4-9-8589-s001.tif]

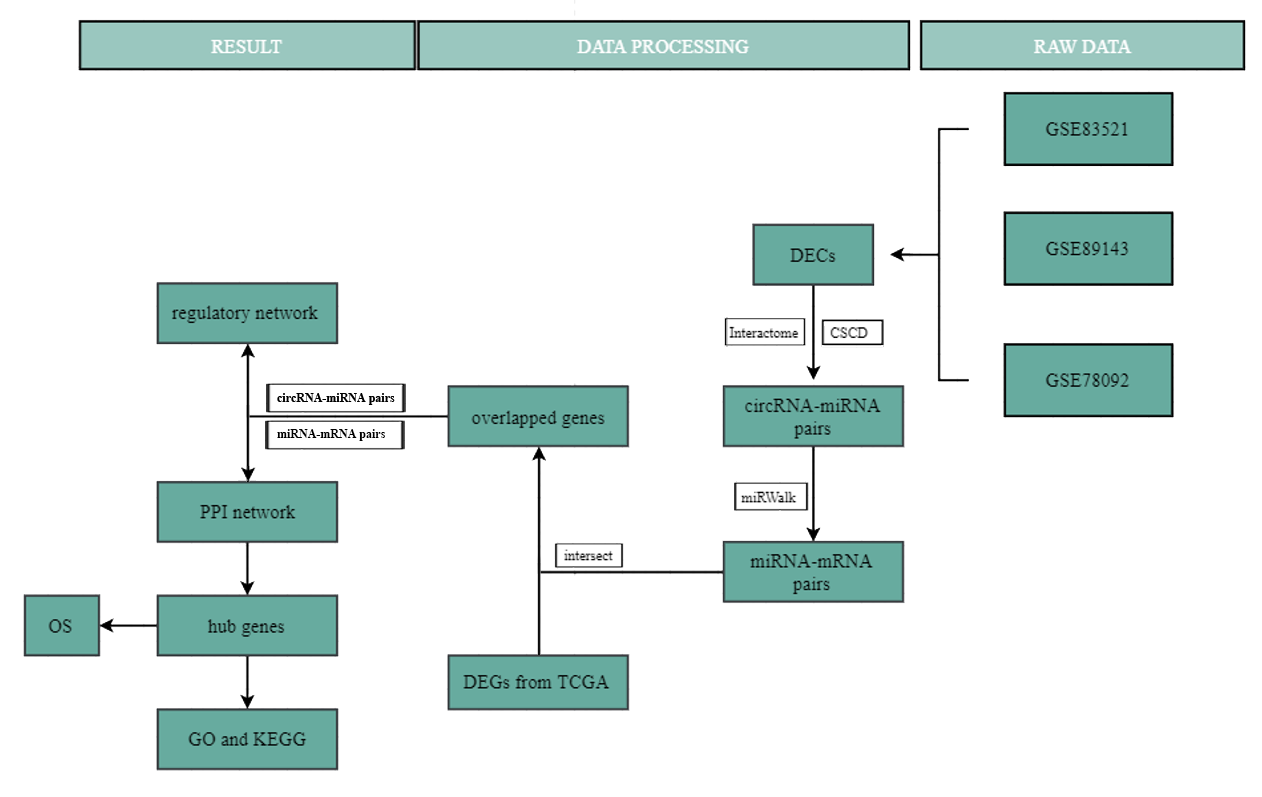

Supplement: Supplementary file 2 — Figure S2 [file CAM4-9-8589-s002.tif]
