# Supplementary material for: Develop a circular RNA–related regulatory network associated with prognosis of gastric cancer
Source: Cancer Med. 2020 Sep 9;9(22):8589–99. doi: 10.1002/cam4.3035 (PMC7666747; doi:10.1002/cam4.3035)
Supplement: Supplementary file 3 — Table S1 [file CAM4-9-8589-s003.doc]

**Table 1 Basic information of the three microarray datasets from GEO**

| Name | Platform | Author | Year | Country | Sample size (T/N) |
| --- | --- | --- | --- | --- | --- |
| GSE83521 | [GPL19978](https://www.ncbi.nlm.nih.gov/geo/query/acc.cgi?acc=GPL19978) | Yan Zhang | 2017 | China | 6/6 |
| GSE89143 | [GPL19978](https://www.ncbi.nlm.nih.gov/geo/query/acc.cgi?acc=GPL19978) | Junming Guo | 2017 | China | 3/3 |
| GSE78092 | GPL21485 | Yousheng Huang | 2016 | China | 3/3 |
