# Supplementary material for: Develop a circular RNA–related regulatory network associated with prognosis of gastric cancer
Source: Cancer Med. 2020 Sep 9;9(22):8589–99. doi: 10.1002/cam4.3035 (PMC7666747; doi:10.1002/cam4.3035)
Supplement: Supplementary file 4 — Supplementary Material [file CAM4-9-8589-s004.docx]

**Retrieval strategy**

#1 (((((((((((((((((("Stomach Neoplasms"[Mesh]) OR Neoplasm, Stomach) OR Stomach Neoplasm) OR Neoplasms, Stomach) OR Gastric Neoplasms) OR Gastric Neoplasm) OR Neoplasm, Gastric) OR Neoplasms, Gastric) OR Cancer of Stomach) OR Stomach Cancers) OR Gastric Cancer) OR Cancer, Gastric) OR Cancers, Gastric) OR Gastric Cancers) OR Stomach Cancer) OR Cancer, Stomach) OR Cancers, Stomach) OR Cancer of the Stomach) OR Gastric Cancer, Familial Diffuse

#2 (((stomach) OR craw) OR tummy) OR gastric

#3 ("Neoplasms"[Mesh]) OR (((((((((((((((((Neoplasia) OR Neoplasias) OR Neoplasm) OR Tumors) OR Tumor) OR Cancer) OR Cancers) OR Malignancy) OR Malignancies) OR Malignant Neoplasms) OR Malignant Neoplasm) OR Neoplasm, Malignant) OR Neoplasms, Malignant) OR Benign Neoplasms) OR Neoplasms, Benign) OR Benign Neoplasm) OR Neoplasm, Benign)

#4 #2 AND #3

#5 #1 OR #4

#6 (CircRNA) OR circular RNA

#7 #5 AND #6 AND "Homo sapiens"[porgn:__txid9606] Sort by: NSAMD Filters: Series

**Inclusion and exclusion criteria**

**1.Inclusion criteria**

1. all of the patients were diagnosed with GC;
2. the studies must contain circRNA expression data both in cancerous and normal gastric tissues;
3. the sample sizes in tumor and non-tumor group were at least three;
4. the samples must be solid tissue samples.

**2.Exclusion criteria**

1. all of the patients were diagnosed with GC;
2. plasma samples, fecal sample, saliva samples, Cell lines or animal experiments;
3. poor sample data quality.
