# Supplementary material for: Develop a circular RNA–related regulatory network associated with prognosis of gastric cancer
Source: Cancer Med. 2020 Sep 9;9(22):8589–99. doi: 10.1002/cam4.3035 (PMC7666747; doi:10.1002/cam4.3035)
Supplement: Supplementary file 5 — Supplementary Material [file CAM4-9-8589-s005.pdf]

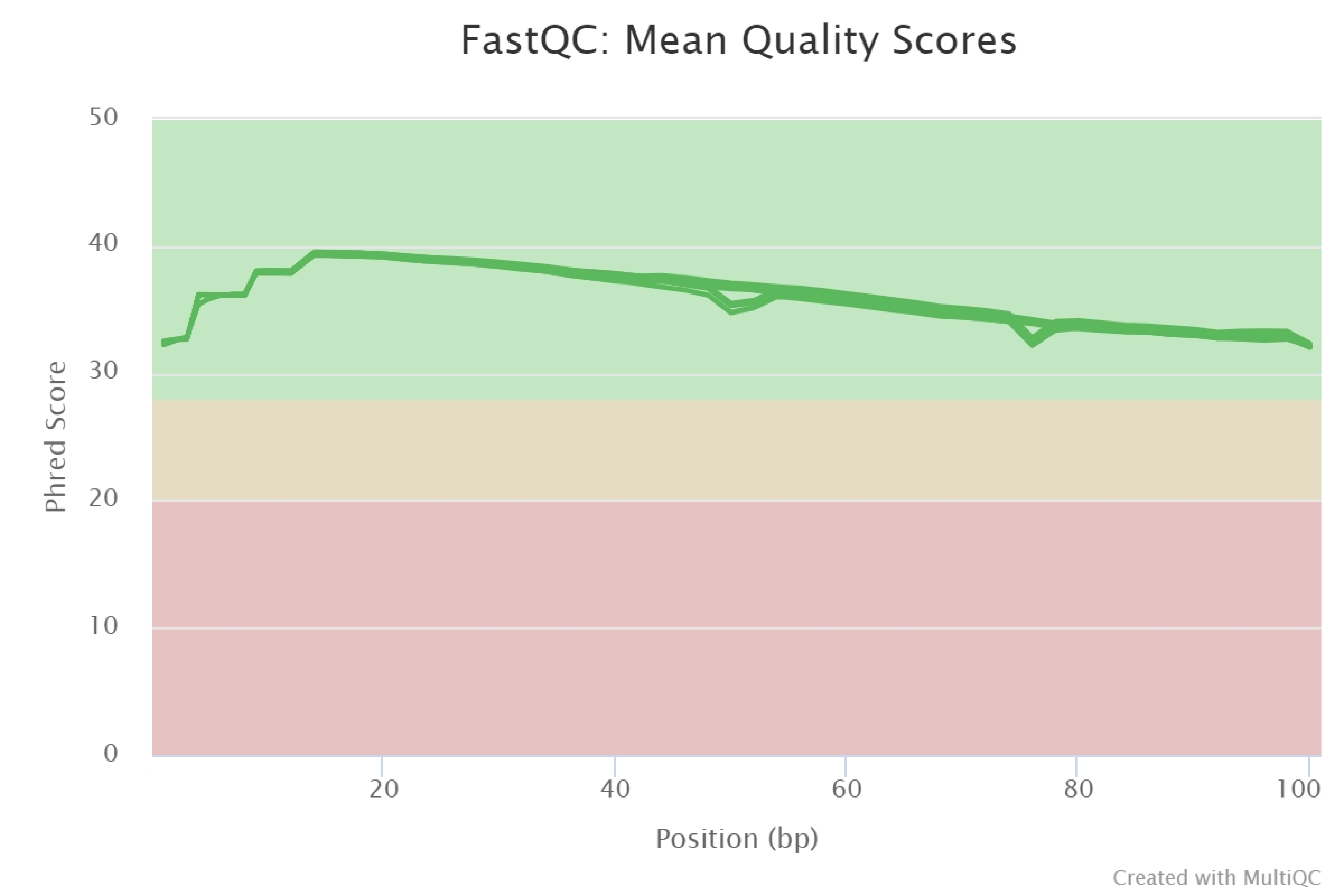
FastQC: Mean Quality Scores
Phred Score
0
10
20
30
40
50
20
40
60
80
100
Position (bp)
Created with MultiQC

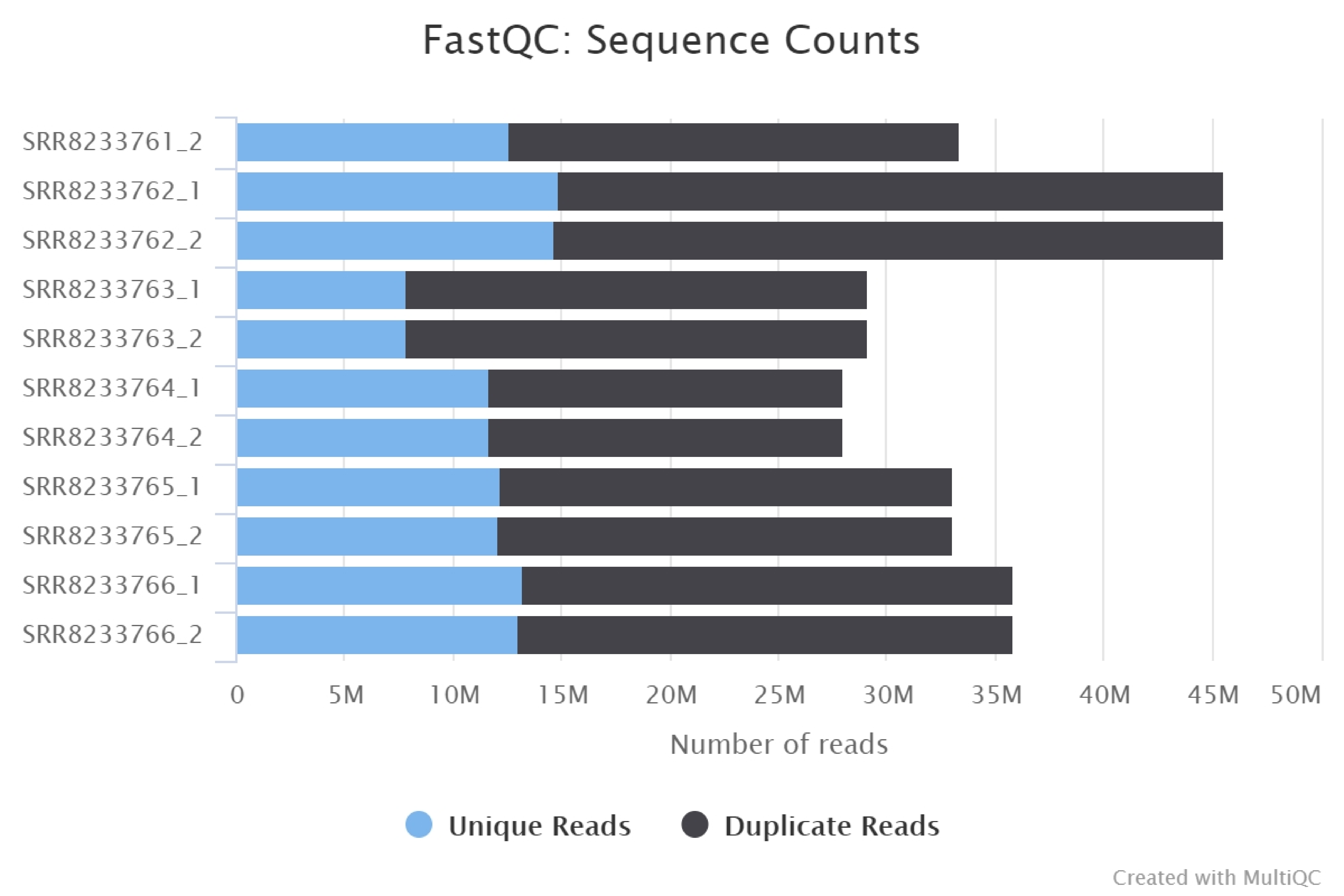
FastQC: Sequence Counts
SRR8233761_2
SRR8233762_1
SRR8233762_2
SRR8233763_1
SRR8233763_2
SRR8233764_1
SRR8233764_2
SRR8233765_1
SRR8233765_2
SRR8233766_1
SRR8233766_2
0
5M
10M
15M
20M
25M
30M
35M
40M
45M
50M
Number of reads
Unique Reads
Duplicate Reads
Created with MultiQC

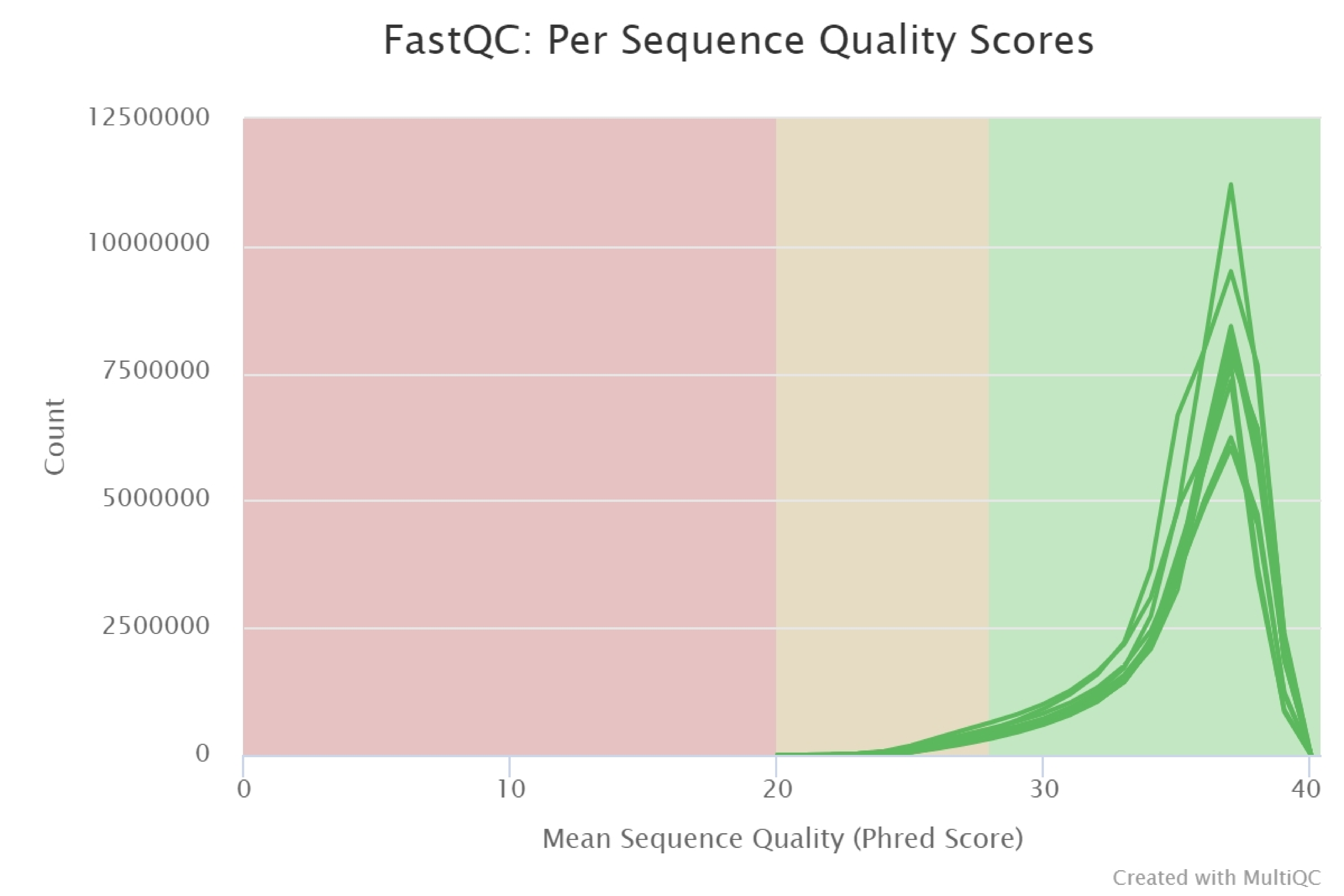
FastQC: Per Sequence Quality Scores
12500000
10000000
7500000
5000000
2500000
0
Count
0
10
20
30
40
Mean Sequence Quality (Phred Score)
Created with MultiQC

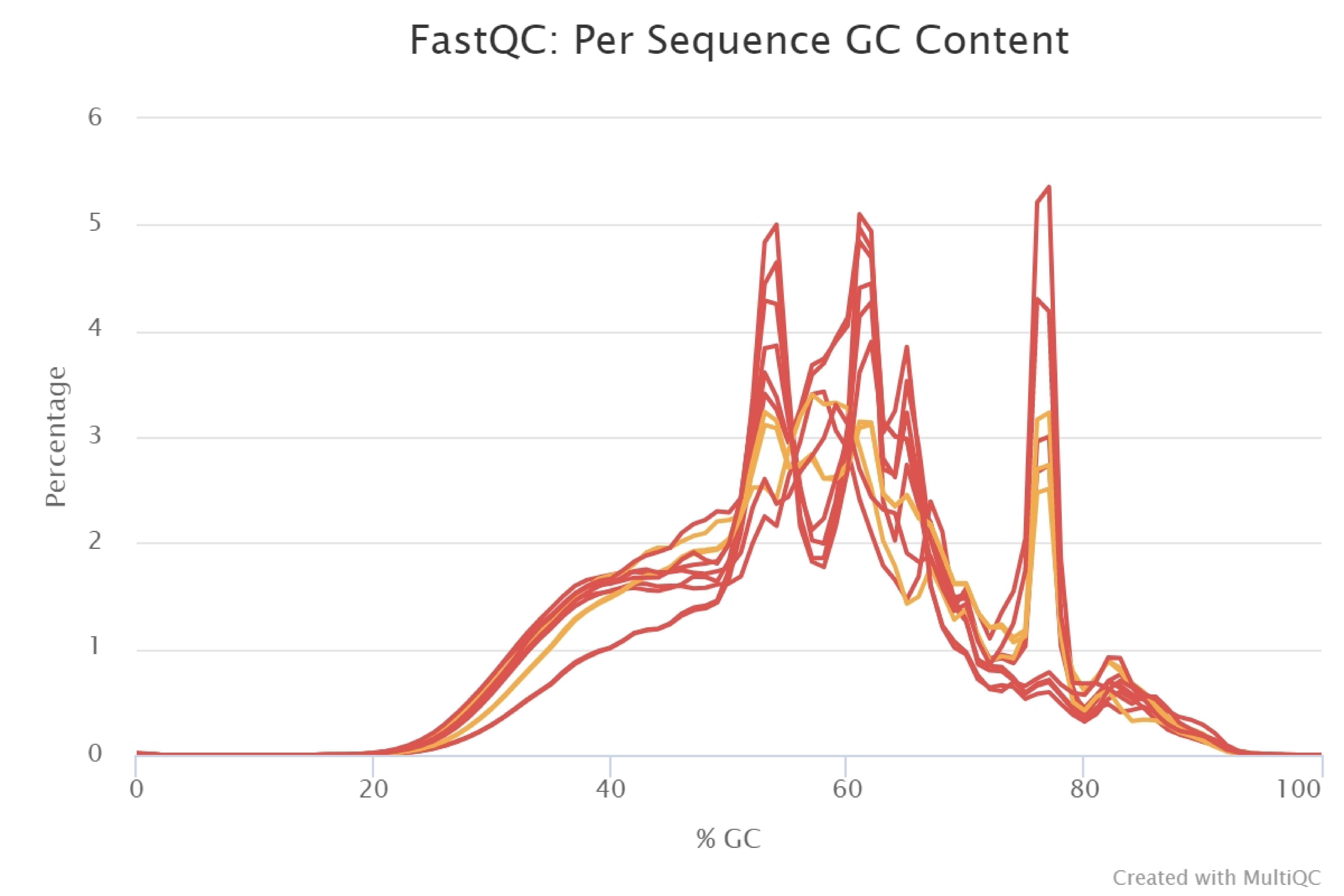
FastQC: Per Sequence GC Content
Percentage
0
1
2
3
4
5
6
0
20
40
60
80
100
% GC
Created with MultiQC

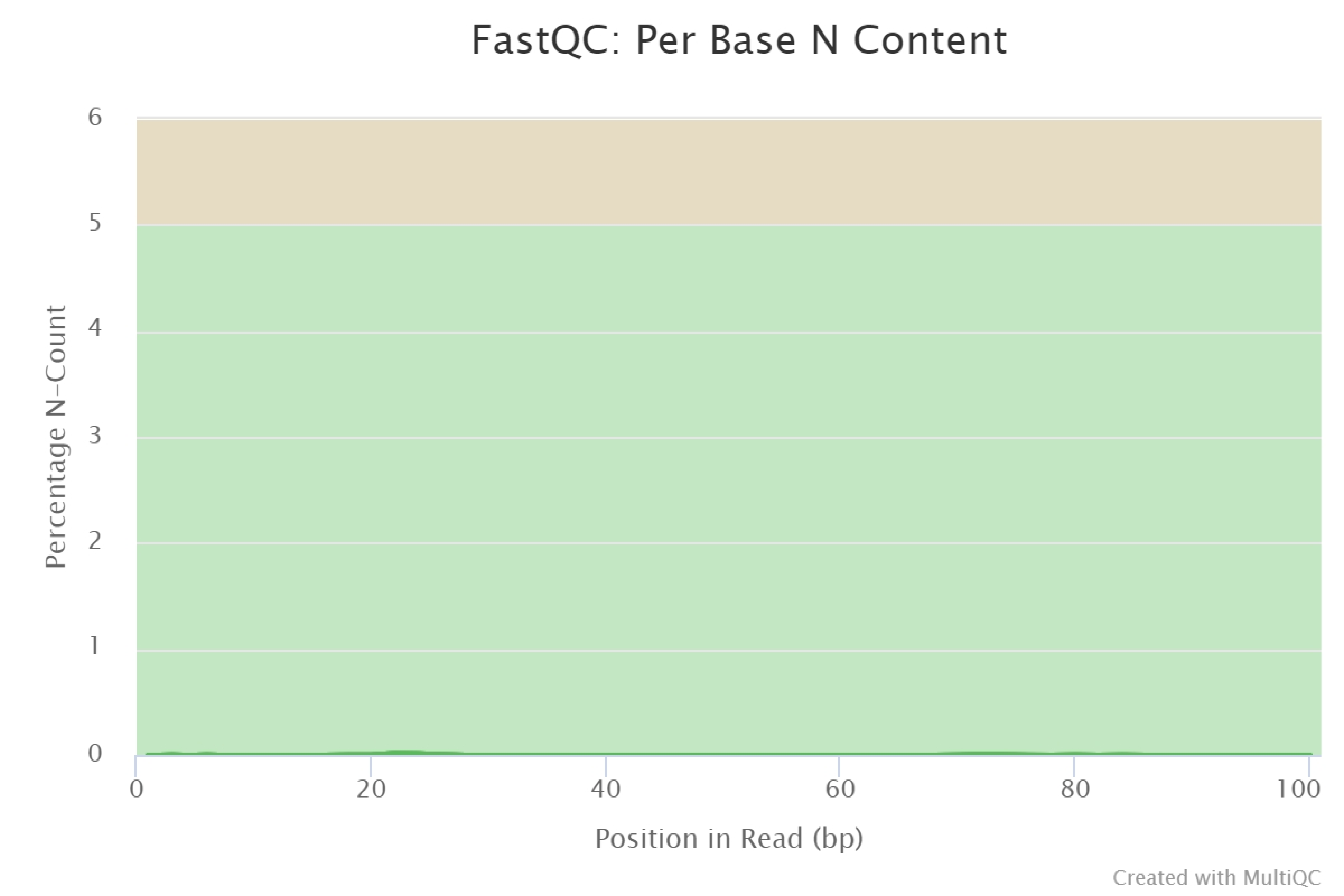

FastQC: Per Base N Content
Percentage N-Count
6
5
4
3
2
1
0
0
20
40
60
80
100
Position in Read (bp)
Created with MultiQC

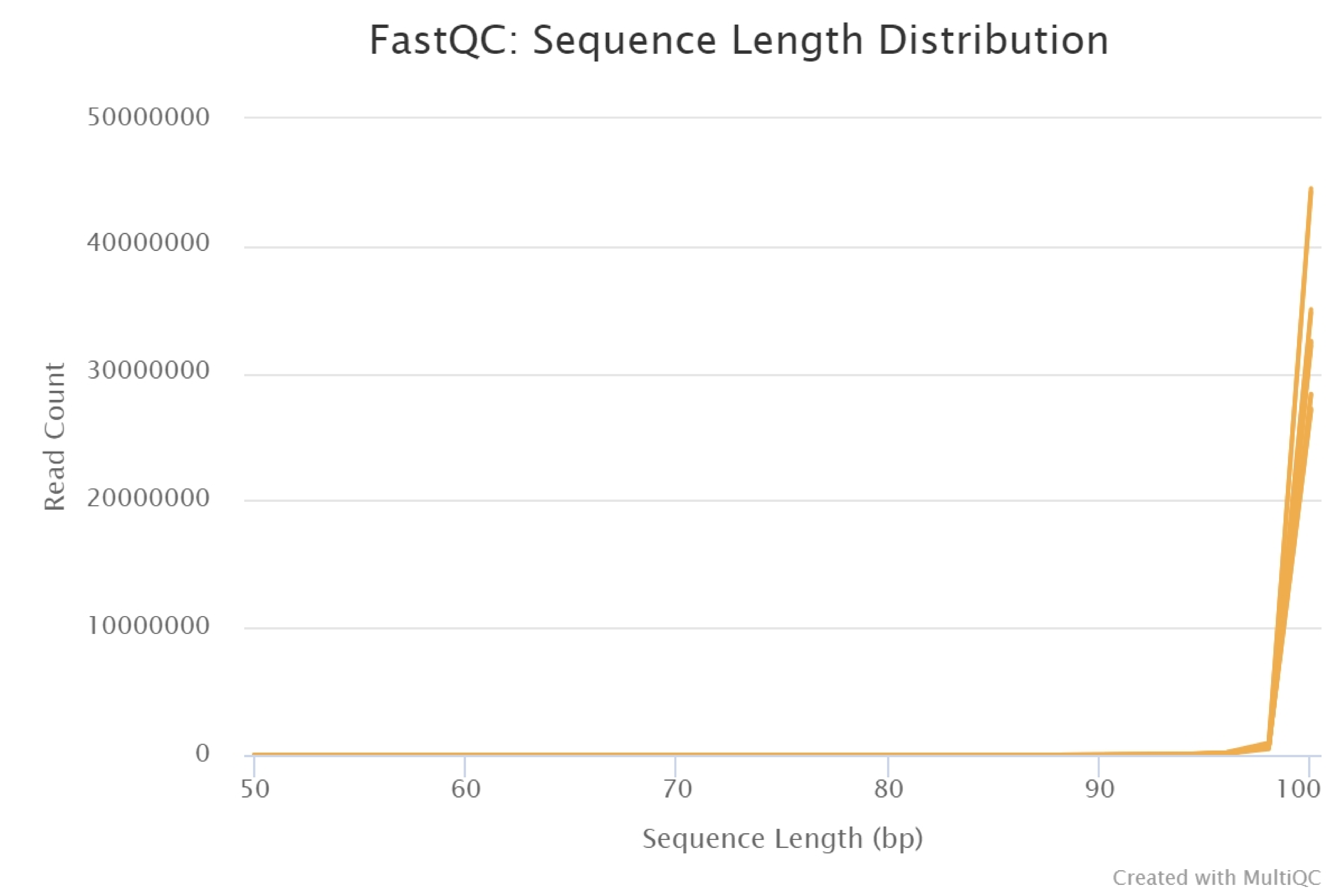
FastQC: Sequence Length Distribution
Read Count
50000000
40000000
30000000
20000000
10000000
0
50
60
70
80
90
100
Sequence Length (bp)
Created with MultiQC

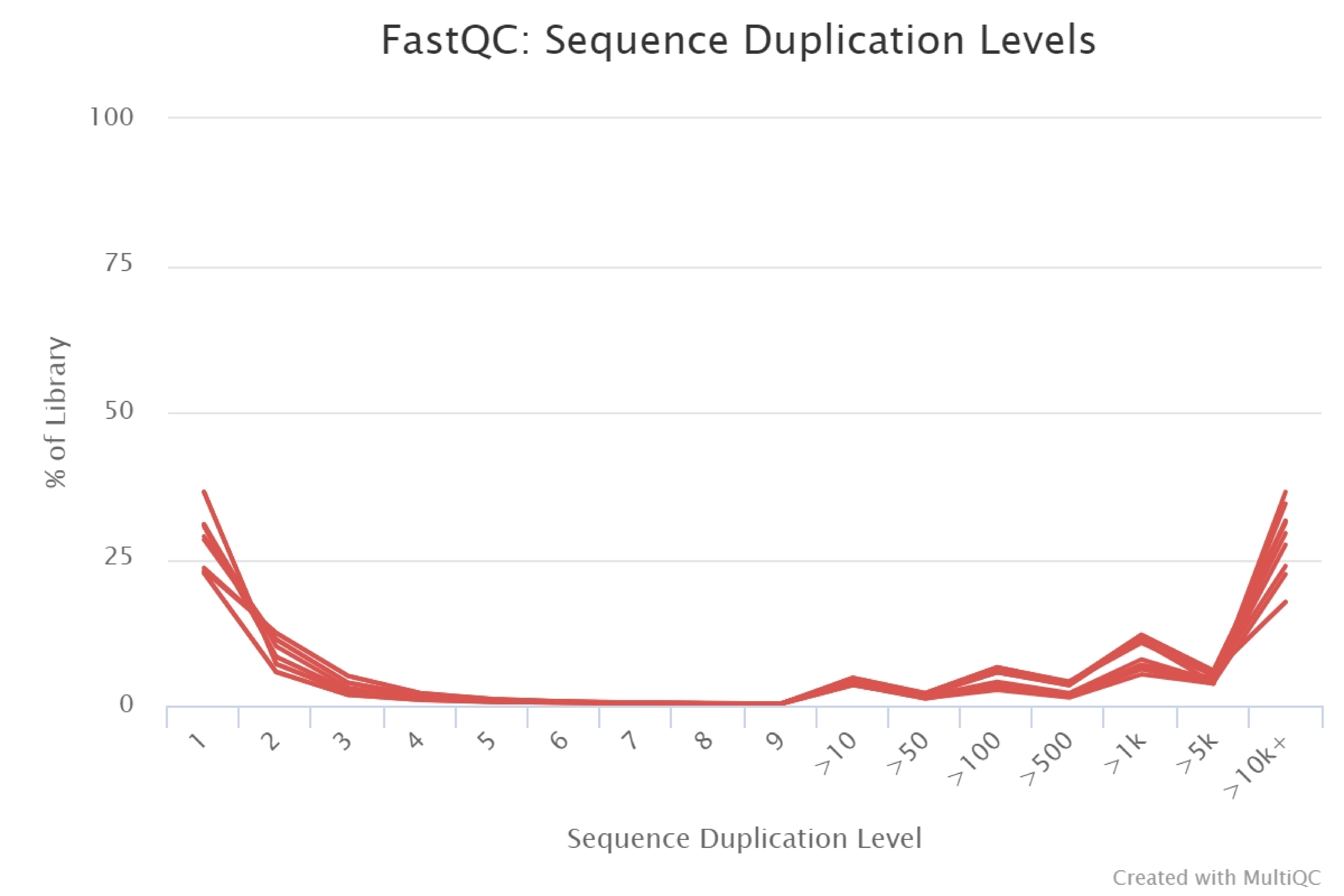
FastQC: Sequence Duplication Levels
100
75
50
25
0
% of Library
1
2
3
4
5
6
7
8
9
>10
>50
>100
>500
>1k
>5k
>10k+
Sequence Duplication Level
Created with MultiQC

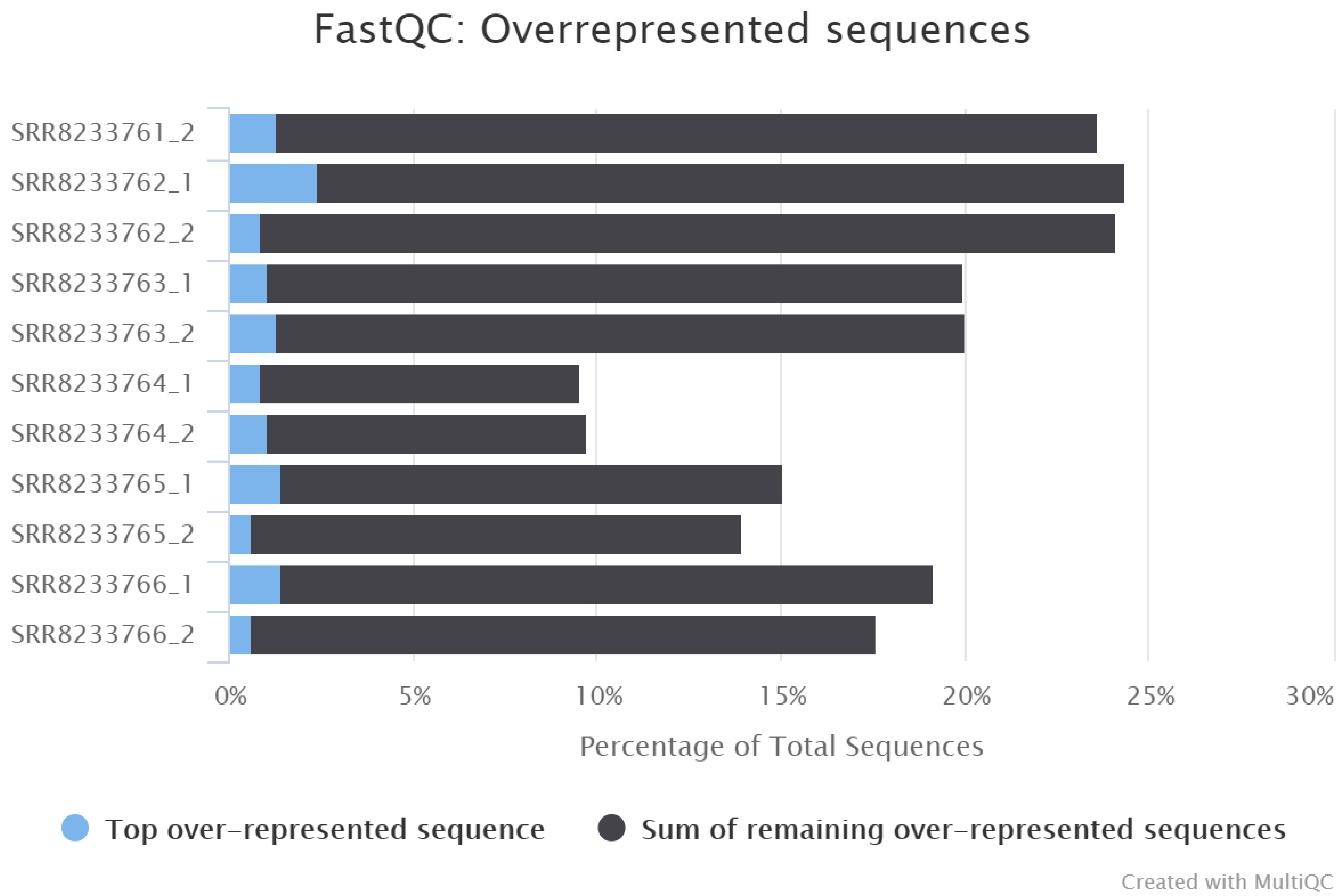

FastQC: Overrepresented sequences
SRR8233761_2
SRR8233762_1
SRR8233762_2
SRR8233763_1
SRR8233763_2
SRR8233764_1
SRR8233764_2
SRR8233765_1
SRR8233765_2
SRR8233766_1
SRR8233766_2
0%
5%
10%
15%
20%
25%
30%
Percentage of Total Sequences
Top over-represented sequence
Sum of remaining over-represented sequences
Created with MultiQC

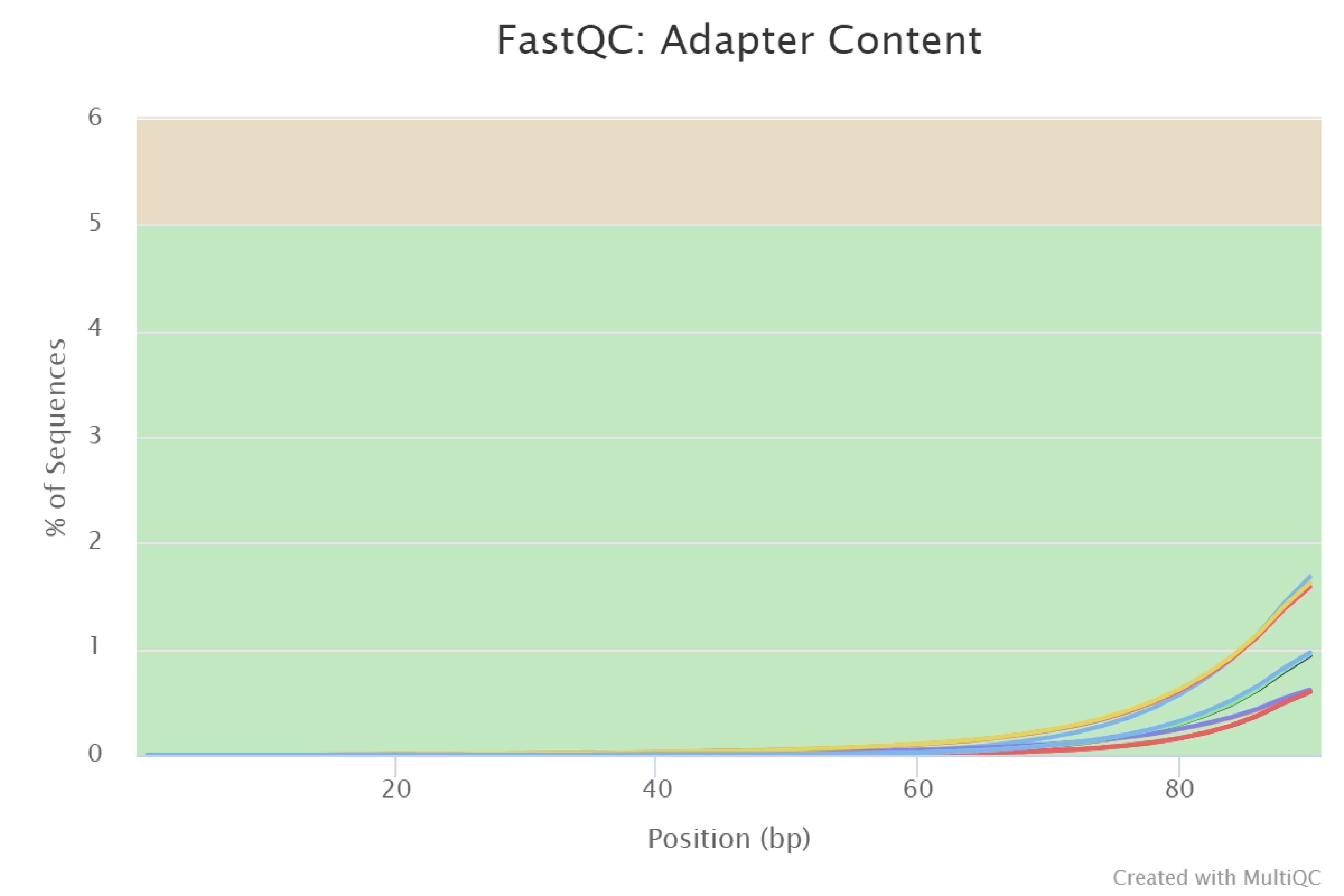
FastQC: Adapter Content
6
5
4
3
2
1
0
% of Sequences
20
40
60
80
Position (bp)
Created with MultiQC
